# Cost and Cost-Effectiveness of the Mediterranean Diet: Results of a Systematic Review

**DOI:** 10.3390/nu5114566

**Published:** 2013-11-18

**Authors:** Rosella Saulle, Leda Semyonov, Giuseppe La Torre

**Affiliations:** Department of Public Health and Infectious Diseases, Sapienza University of Rome, Piazzale Aldo Moro 5, Rome 00185, Italy; E-Mails: rosella.saulle@uniroma1.it (R.S.); leda.semyonov@uniroma1.it (L.S.)

**Keywords:** Mediterranean diet, systematic review, cost-analysis, cost-effectiveness, cost-utility, cost-benefit

## Abstract

The growing impact of chronic degenerative pathologies (such as cardiovascular disease, type 2 diabetes and Alzheimer’s disease) requires and pushes towards the development of new preventive strategies to reduce the incidence and prevalence of these diseases. Lifestyle changes, especially related to the Mediterranean diet, have the potential to modify disease outcomes and ultimately costs related to their management. The objective of the study was to perform a systematic review of the scientific literature, to gauge the economic performance and the cost-effectiveness of the adherence to the Mediterranean diet as a prevention strategy against degenerative pathologies. We investigated the monetary costs of adopting Mediterranean dietary patterns by determining cost differences between low and high adherence. Research was conducted using the PubMed and Scopus databases. Eight articles met the pre-determined inclusion criteria and were reviewed. Quality assessment and data extraction was performed. The adherence to the Mediterranean diet has been extensively reported to be associated with a favorable health outcome and a better quality of life. The implementation of a Mediterranean dietary pattern may lead to the prevention of degenerative pathologies and to an improvement in life expectancy, a net gain in health and a reduction in total lifetime costs.

## 1. Introduction

The Mediterranean diet has been widely reported as a model of healthy eating, for its contribution to a favorable healthy status and for numerous health benefits, including its inverse relationships with cardiovascular diseases (CVDs) and metabolic syndrome (a health condition characterized by abdominal obesity, dyslipidemia, elevated blood pressure and impaired glucose tolerance) [1,2,3,4,5].

In addition, many investigators have already underlined the beneficial role that this dietary pattern may have in coagulation processes and inflammation, since it provides a significant source of antioxidant vitamins [6,7].

According to the original definition of Keys [8], the typical Mediterranean diet is characterized by high consumption of olive oil (as the prevalent visible fat), vegetables, legumes, whole-grain products, fruits and nuts. The intake of saturated animal fats is relatively low, and there is a moderate fish consumption (depending on the proximity to the sea), which furnishes enough provision of polyunsaturated fats, thus making it a low-glycemic-index diet [9].

The recent recognition by the United Nations Educational, Scientific and Cultural Organization (UNESCO) of the Mediterranean diet as an Intangible Cultural Heritage of Humanity reinforces, together with the scientific evidence, the Mediterranean diet as a cultural and health model [10,11].

Since the 1960s, the Seven Countries Study [12] showed that populations in the Mediterranean region experienced lower cardiovascular disease (CVD) mortality compared with northern European populations or the US, probably as a result of different dietary patterns. Later observational studies have confirmed the benefits associated with an adherence to a Mediterranean dietary pattern on CVD risk factors [13].

The healthcare costs attributable to diseases and conditions related to nutrition are large, as already suggested in a “quite old” study in 1998, with estimates of medical expenses for overweight/obesity alone in the U.S. totaling $78.5 billion or 9.1% of total medical expenditure [14].

Nowadays, cardiovascular disease is one of the leading causes of mortality and morbidity in many industrialized countries [15].

There has been a significant change in dietary habits and physical activity levels worldwide as a result of industrialization, urbanization, economic development and food market globalization [16].

Diseases and conditions linked to an unhealthy diet include cardiovascular disease (29.2% of global deaths), diabetes (171 million people worldwide) and cancer (12.5% of global deaths) [16]. Obesity has reached alarming proportions, and it is estimated that at least 300 million adults are clinically obese [16,17,18,19,20].

Consequently, the growing impact of chronic degenerative pathologies (such as cardiovascular and respiratory disease, stroke, type 2 diabetes and Alzheimer’s disease, hypertension, dyslipidemia and cancers) in high income countries requires and pushes towards the development of new preventive strategies to reduce the incidence and prevalence of these diseases.

As far as the economic aspect is concerned, the cost of coronary heart disease (CHD) and chronic diseases includes the direct costs of physicians’ and other healthcare professionals’ salaries, the costs of hospital nursing, home services and medication and some indirect costs associated with reduced productivity due to illness and disability.

Lifestyle changes especially related to the Mediterranean diet have the potential to modify disease outcomes and, therefore, the costs associated with their management.

However, food choice is strongly influenced by economic constraints. Cade *et al*. [21] showed that a high adherence to a healthy dietary pattern is usually associated with higher monetary costs. Furthermore, several studies have shown that economic constraints lead to the consumption of less healthy diets that are characterized by high energy density and palatability [22,23,24].

In the literature, there is a scarcity of studies evaluating the relationship between food costs and adherence to different food patterns.

The objective of this study was to perform an economic evaluation through a systematic review of the scientific literature, to gauge the cost-effectiveness of an adherence to the Mediterranean diet as a prevention strategy for degenerative pathologies through assessing the economic performance of this diet, by investigating the monetary costs of the adoption of this dietary pattern and by determining the cost differences between low and high adherence to it.

## 2. Materials and Methods

### 2.1. Identification of Relevant Studies

A literature review was conducted using two electronic medical journal databases: Scopus and PubMed engines for published studies on economic evaluations of Mediterranean diet adherence.

The keywords used were “Mediterranean diet”, “cost effectiveness”, “cost utility”, “cost benefit” and “cost”. Combined searches were carried out for: “Mediterranean diet AND cost effectiveness”, “Mediterranean diet AND cost utility”, “Mediterranean diet AND cost benefit” and “Mediterranean diet AND cost”.

**Figure 1:**
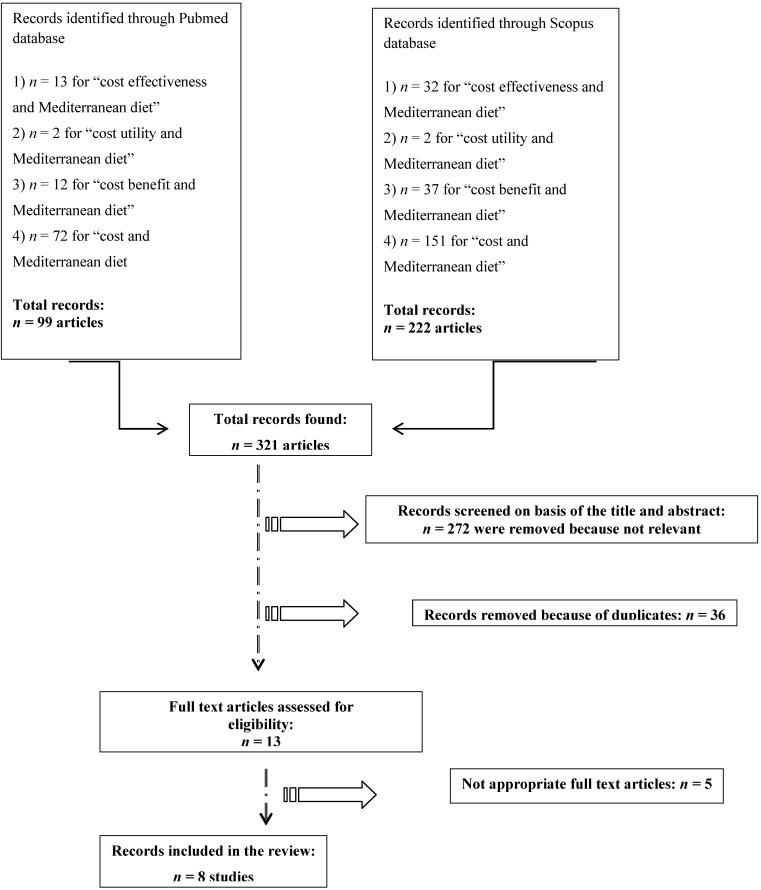
Flow-chart of the selection process.

Search criteria are summarized in Figure 1.

We applied no date restrictions to the database search, but the selection of the study was limited to articles published in the English and Italian language. We selected all studies that focused on the economic evaluation of Mediterranean diet adherence without the limit of population or country and including all study designs.

All the review process, search and selection (identification, screening, eligibility of included studies) were performed according to the PRISMA criteria [25] (Figure 1).

In the selection process, abstracts were initially read independently by two researchers who identified potentially eligible full text papers, which were then retrieved and assessed in order to decide on their final inclusion. Pertinent references included in the articles were reviewed, too, and when the cited papers contained outcomes related to economic evaluation concerning the Mediterranean diet, they were selected and included in our review.

Articles were examined and were included if:

(1) The research was based on modeling the impact of Mediterranean diet adherence on the epidemiology of CVD or other chronic disease.
(2) Studies evaluated the impact of adopting a Mediterranean diet on individual dietary cost and the monetary costs for Mediterranean food-intake patterns (for example, the cross-sectional survey based on the evaluation of the monetary costs for all food items of the administered food frequency questionnaire (FFQ) calculated by multiplying the amount of food consumed from the FFQ with average national prices, *etc.*).

Articles were excluded if:

(1) Studies did not refer to the Mediterranean diet concerning economic evaluation outcomes.
(2) The full text was not available.
(3) Economic data were not reported.

When Medline outcomes and references cited in the studies overlapped, all duplicate articles were removed.

### 2.2. Quality Assessment and Data Extraction

For each selected study, two researchers (R.S. and L.S.) independently assessed its quality according to Drummond’s original checklist [26] (Table 1) as modified by La Torre *et al*. [27], weighted median scores for each item by different experts.

**Table 1:** Score quality of the cost-effectiveness analysis of the included studies by year of publication (according to Drummond’s checklist, as modified by La Torre *et al*. [27]).

Authors (Year of Publication)	Dalziel K. *et al*. [28], 2006	Panagiotakos D. *et al*. [29], 2007	Dalziel *et al*. [30], 2007
Study design Score/Items (26 score items)	26/26	23/26	26/26
Data collection Score/Items (45 score items)	28/45	22/45	45/45
Analysis and interpretation of results Score/Items (48 score items)	39/47	28/47	47/47
Total Score/Items (119 total score)	93/118 = 78.81%	73/118 = 61.86%	118/118 = 100%

Discrepancies between the two investigators were solved by oral discussion and consensus with a senior investigator (G.L.T.). Each item was assigned with the median weight attributed by consensus, if applicable. Finally, the global score was computed by summing weights of each item. To compare different studies, global scores were referred to in percentages, to the highest score achievable with the weighted Drummond’s checklist.

Drummond’s checklist is composed of 35 items divided into 3 sections: study design, data collection and analysis and interpretation of results. To “weight” the items, a group of experts was asked to attribute a score according to its importance. The weighted scores that were assigned by consensus to the study design, data collection and analysis and interpretation of results were 26, 45 and 48, respectively. For each item section, the maximum achievable score was as follows:

1. Study design (7 items): maximum global score = 26;
2. Data collection (14 items): maximum global score = 45;
3. Analysis and interpretation of results (14 items): maximum global score = 48.

When the item was not applicable to the study, we reduced the maximum global score from the relative weighted score item.

Two reviewers used a data collection to form independently abstract data from the studies. The information extracted were: references, publication year and type of analyses, alternatives, nation/perspective, sample, efficacy measures/cost measure and results. The reviewers discussed any discrepancies in their results until agreement was reached. The characteristics of each study are shown in Table 2 and Table 3.

## 3. Results

### 3.1. Identification of Relevant Research

Not applying language restrictions nor temporal time limitation, we found a total of 99 articles from the PubMed search and 222 articles from the Scopus search.

A total of 321 articles were found for all strings. We included all study designs, taking into account the review article, too, by which we extracted all pertinent literature references.

Furthermore, articles were subjected to an accurate screening of both the title and the abstract from the two search engines separately and to the removal of duplicates in each database.

Of the 321 articles, 272 were removed, because they were not relevant (lack of data, researched other Mediterranean diet outcomes data or simply because they did not correspond to our objective) and 36 articles were duplicates. Finally, 13 articles were assessed for eligibility: all full texts were examined; at the end of the evaluation, five were excluded, because they had no data on economic evaluation or did not have appropriate full text articles. One of the five articles conducted by Peracino *et al*. [31] in their Appendix simply reported data from the study conducted by Dalziel *et al*. [28], which had already previously been selected in the database search and, therefore, already included in the review, so the article conducted by Peracino *et al*. [31] was excluded by the review. At the end of the selection process, only eight articles met the pre-determined criteria described above [28,29,30,32,33,34,35,36].

The reference sections of all eight articles were examined in order to achieve other pertinent studies, but the interesting cited references that we selected, which reported economic evaluation data, were overlapped in the eight selected studies. The included studies had two different targets: (a) one type of study was based on the cost-effectiveness analysis or on modeling of the impact of the Mediterranean diet adherence on the epidemiology of CVD or other chronic disease; (b) the other type of study was interested in the micro-costing and direct and indirect costs derived from studies that evaluated the impact of adopting a Mediterranean diet on individual dietary cost and the monetary costs for foods relevant to the Mediterranean lifestyle pattern (for example, the cross-sectional survey based on the evaluation of the monetary costs for all food items of the administered food frequency questionnaire (FFQ)).

**Table 2:** Characteristics of the selected studies by year of publication and Mediterranean diet micro-costing analysis. FFQ, food frequency questionnaire; CVD, cardiovascular disease.

References	Study Design	Type of Analyses	Diseases Outcomes	Alternatives	Nation/Perspective	Sample	Efficacy Measures/Cost Measures	Main Results
Vlismas *et al.* [32], 2009	Cross-sectional	Micro costing analysis: - Dietary cost; - Cost of individual food items (FFQ)	Cardiovascular disease	Mediterranean diet: adherence through a semiquantitative FFQ (Mediterranean diet score)	Greece	1514 men and 1528 women (aged > 18 years) without known CVD	Direct cost Micro-costing analysis	The weekly cost of participants’ diets varied from 5.35 to 83.57 €/week in men (mean 25.45 (SD 6.80) €/week) and from 10.89 to 55.49 €/week in women (mean 25.63 (SD 6.30) €/week).
Drewnowski *et al.* [33], 2009	Narrative review	Micro costing analysis: - Dietary cost - Cost of individual food items	None	Mediterranean diet North American modified version of Mediterranean diet	USA	None	Direct cost Micro-costing analysis	Mediterranean-style foods can be obtained at all price ranges, whether calculated per 100 g or per 4.18 MJ (1000 kcal). Generally, nuts and seeds cost between $1.00–25.00 per 4.18 MJ (1000 kcal), in part due to their relatively high energy density. Legumes represent an important protein source, while offering relatively low energy density at a relatively low cost ($1.00: 25.00 per 4.18 MJ or $1.00: 25.00 per 1000 kcal).
Goulet *et al.* [34], 2007	Cohort survey	Micro-costing analysis: - Dietary cost - Costs of individual food items (FFQ) to obtain total dietary cost	None	Adherence/non-adherence to Mediterranean diet: a partial score varying from 0 to 4. Dietary cost at week 0, 12 and 24 of the nutritional intervention promotion of the adoption of a Mediterranean food pattern in women who did or did not plan their food purchases as a function of weekly discounts.	Canada-Quebec City	A total of 126 healthy women aged 30–65 years were invited to a screening visit for an evaluation of their food habits. 74 women completed the nutritional intervention.	Direct cost Micro-costing analysis	Energy density, energy cost, total dietary cost and daily cost of specific food items at week 0, 12 and 24 total dietary cost nor energy cost at week 12 or 24 in the nutritional intervention promoting the adoption of a Mediterranean food pattern in women changed significantly from baseline. **Energy cost, kJ/Canadian dollars (CAN$**): 1046 ± 217 (week 0); 967 ± 162 (week 12); 967 ± 192 (week 24) **Total daily dietary cost, (CAN$): ** 1046 ± 217 (week 0); 967 ± 162 (week 12); 967 ± 192 (week 24). The changes between week 0 and 12 in total daily dietary cost differed between women who planned and those who did not plan food purchases based on weekly discounts*.* **Dietary cost for planned: (CAN$)/day: ** 9.31 ± 2.76 (week 0); 8.67 ± 2.15 (week 12); 9.17 ± 2.08 (week 24) **Energy cost, kJ/(CAN$): ** 1040 ± 215 (week 0); 975 ± 184 (week 12); 951 ± 167 (week 24) **Dietary cost for did not plan: (CAN$)/day** 8.08 ± 1.99 (week 0); 8.54 ± 2.14 (week 12); 8.45 ± 2.26 (week 24) **Energy cost for did not plan: kJ/CAN$: ** 1052 ± 222 (week 0); 957 ± 131 (week 12); 988 ± 220 (week 24). It has been suggested that the diet of low income consumers, for whom food price is a key factor in determining food choice, may be high in sugar and fat simply because these are the cheapest sources of dietary energy available.
Lopez *et al.* [35], 2009	Cohort survey	Micro-costing analysis: - Dietary cost - Costs of individual food items (FFQ) to obtain total dietary cost	None	Mediterranean diet Western diet	Spain	19,057 subjects with a mean age of 38.6 (SD 12.2) years and comprised of 60% women. After excluding for extremes of caloric intake (800 or 4000 kcal/day for men and 500 or 3500 kcal/day for women) (*n* = 1700) and biologically implausible values for height and weight (*n* = 160), 17,197 subjects remained. These subjects were analyzed for the association between costs and dietary pattern.	Direct cost Micro-costing analysis	A higher score on the Mediterranean dietary pattern was positively associated with increased costs of daily food consumption after adjusting for age and sex, whereas a higher score on the Western dietary pattern was inversely associated with cost. A healthy Mediterranean dietary pattern is more expensive (fifth quintile *vs*. first quintile of scores of adherence: +€0.71 (+$0.90) (95% CI: +€0.67 to +€0.74) per 1000 kcal) than a Westernized dietary pattern (fifth quintile *vs*. first quintile of scores of adherence: −€0.64 (−$0.80) per 1000 kcal (95% CI: −€0.68 to −€0.61) after adjusting for potential confounders among healthy middle-aged Spanish graduates. After adjusting for dietary pattern scores and other potential confounders, higher daily food costs were significantly associated with greater weight gain. Mediterranean dietary pattern is more expensive to follow than a Western dietary pattern. This economic barrier should be considered when counseling patients about following a healthy diet, because cost may be a prohibitive factor.
Schroder *et al.* [36], 2006	Cross-sectional survey	Micro-costing analysis: - Dietary cost - Costs of individual food items (FFQ) to obtain total dietary cost; Average food prices were calculated.	Body mass index (BMI) and obesity	Mediterranean diet: Mediterranean Diet Score (MDS) and the Healthy Eating Index (HEI)	Spain	Six thousand free-living Spanish men and women, aged between 25 and 74 years, were randomly selected from the general population of Girona, according to the 1996 census, and participated in this study from 1999 to 2000. After excluding census errors, 4359 eligible subjects were left, of whom 3179 agreed to participate.	Direct cost Micro-costing analysis	An increase in 1 Euro ($1.25) of monetary diet costs per day was associated with a change of 0.46 units (*P* < 0.001) and 2.03 units (*P* < 0.001) in the MDS and HEI, respectively. The magnitude of the association was similar to both scores after standardization. Subjects who closely adhered to the MDS and HEI paid 1.2 Euro ($1.50) (*P* < 0.001) and 1.4 Euro ($1.75) (*P* < 0.001) daily more for food consumption, respectively, than those who weakly adhered to these dietary patterns. Energy- and age-adjusted analysis revealed that subjects with a high adherence to the MDS and HEI paid 7.92 Euro ($9.90) and 8.14 Euro ($10.2), respectively, for their daily food consumption. In contrast, a low adherence to the MDS and HEI was associated with a daily monetary cost for food consumption of 6.74 Euro ($8.43) and 6.76 Euro ($8.45), respectively. The most important result of the present study was that monetary diet costs increased with higher adherence to the MDS and HEI. Furthermore, both dietary patterns were inversely associated with BMI and obesity after controlling for several confounders.

**Table 3:** Characteristics of the selected studies by year of publication and types of economic analysis. CUA, cost-utility analysis. CEA, cost-effectiveness analysis; QALY, quality-adjusted life year.

References	Study Design	Type of Analyses	Diseases Outcomes	Alternatives	Nation/Perspective	Sample	Efficacy Measures/Cost Measures	Main Results
Dalziel *et al.* [28], 2006	RCTs (randomized controlled trial)	CUA	Myocardial infarction	Mediterranean diet over a time frame of 10 years for patients after a first acute myocardial infarction (AMI). A prudent Western diet over a time frame of 10 years for patients after a first acute myocardial infarction (AMI).	France societal perspective healthcare system	605 patients aged >70 years that had survived a myocardial infarction within 6 months of enrolment.	Direct and indirect cost; CER (cost-effectiveness ratio); BCR (Benefit-cost ratio)	The cost-effectiveness results of the Mediterranean diet compared with a prudent Western diet, based purely on the trial results, led to a cost per cardiac death or AMI averted ranging from AU $10,879 (U.S. $7552, €6217), when program and food costs were included, to AU $1778 (U.S. $1234, €1016) when the costs of cardiac events were also incorporated. When modeled over 10 years, the Mediterranean diet resulted in an incremental cost per QALY gained of AU $1013 (U.S. $703, €579) per person. It also led to mean gains of 0.31 life years per person or 0.40 quality adjusted life years per person. One-way sensitivity analyses showed that the Mediterranean diet remained highly cost-effective under all scenarios. The model was most sensitive to the cardiac event rates, the costs of the intervention and the time horizon of the model. The Mediterranean diet dominated (cheaper and more effective) the Western diet under 1 scenario, *i.e.*, when food costs were excluded from the analysis. The estimated cost per QALY gained ranged from AU $417 (U.S. $289, €238) when the number of consultations was halved to AU $7149 (U.S. $4963, €4085) when the lower limit for the intervention effect (more cardiac events) was used.
Panagiotakos *et al.* [29], 2007	Cohort survey, original article (CEA)	CEA	Cardiovascular disease	Mediterranean diet “Westernized” diet	Greece	1514 adult men and 1528 women, without any clinical evidence of cardiovascular disease.	Direct and indirect cost CER (cost-effectiveness ratio) ICER (incremental cost-effectiveness ratio)	Total healthcare cost was estimated to be €336.720 in those who were “away” and €35.880 in those who were closer to this diet pattern. Life-years lost due to disability was 6.8 in those who were “away” and 0.9 in those “close” to this pattern. The incremental cost-effectiveness ratio was €50.989.
Dalziel *et al.* [30], 2007	Review	CEA CUA		Mediterranean diet Intensive lifestyle change (nutrition and physical activity) to prevent diabetes Reduced fat diet for persons with IGT( Impaired Glucose Tolerance) Nutritional counseling in GP (GP, general practice/primary care) Nurse counseling in GP Oxcheck nurse health checks in GP Gutbusters Workplace (for men) Talking computer Multimedia 2 fruit 5 veg campaign the FFFF (Fighting Fit, Fighting Fat) media campaign	Australia/societal perspective	Mediterranean diet: *N* = 303, mean age 54, 9% female; Control: *N* = 302, prudent Western diet (reference reported: de Lorgeril *et al.* [37], 1999)	Cost-effectiveness CER (cost-effectiveness ratio) QALY (quality-adjusted life year)	In relation to major disease outcomes, the Mediterranean diet had cost-effectiveness ratios of AU $3300 (U.S. $2500, £1300) per non-fatal AMI averted and AU $5300 (U.S. $4000, £2100) per death averted. Mediterranean diet intervention for persons after AMI and intensive lifestyle change to prevent diabetes perform exceptionally well: AU $1020 (U.S. $760, £410) and AU $1880 (U.S. $1410, £750) per QALY gained, respectively. Some interventions, notably the Mediterranean diet and the two interventions to prevent type 2 diabetes, as well as Gutbusters Workplace, are cost-saving under plausible sets of assumptions. The Mediterranean diet and intensive lifestyle change to prevent diabetes are the most certain and cost-effective interventions based on good quality trials.

CUA: cost-utility analysis, CEA: cost-effectiveness analysis, CER: cost-effectiveness ratio, BCR: Benefit-cost ratio, ICER : incremental cost-effectiveness ratio, QALY:quality-adjusted life year.

### 3.2. Type of Economic Evaluation of the Included Studies

According to the study design, there were two reviews [28,30], one being a cost-effectiveness analysis (CEA) in a cohort survey [29]. All these three articles evaluated the cost-effectiveness analysis (CEA) [28,30]. In addition, a cost-utility analysis (CUA) was considered in the study carried out by Dalziel 2007 [30], and a cost-benefit analysis (CBA) was considered in the study carried out by Dalziel *et al*. 2006 [28].

The review carried out by Dalziel *et al*. [30] had the specific aims of conducting: (a) a cost-effectiveness analysis based purely on trial results, with performance expressed as cost/natural unit, such as the cost per additional serving of fruit and vegetables per person per day; and where possible (b) modeled cost-utility analysis expressed as cost per quality-adjusted life year (QALY). The other review, carried out by the same author, Dalziel *et al*. [28], had the aim of assessing the economic performance of the Mediterranean diet after myocardial infarction, in terms of cost per quality-adjusted life year (QALY). By expressing performance as cost per QALY, they compared this nutritional intervention with other approaches for the prevention and management of disease. Cost-effectiveness analyses and sensitivity analyses all showed that the Mediterranean diet remained highly cost effective under all scenarios.

The study carried out by Panagiotakos [29] assessed the Mediterranean diet in relation to coronary heart disease risk and its economic consequences, the relationship between the Mediterranean dietary pattern with the 10-year risk of developing CHD (from the cross-sectional study “The ATTICA Study” that is a health and nutrition survey, which is being carried out in the province of Attica) compared to a non-Mediterranean diet, as well as its economic consequences. The analysis was based on a sample of 3042 apparently healthy adults from the general population in Greece. The authors made an assumption that participants with 10-year coronary risk greater than 10% were considered to be prone to experience an event and hospitalized during the next 10 years (*i.e.*, effect). Then, they considered the cost for hospitalization of participants who were “closer” to the Mediterranean diet (*i.e.*, above the median score) and those who were “away” from this dietary pattern. The main measures of effectiveness were (a) the time free from the development of CHD-related end points (*i.e.*, people with 10-year coronary risk 10%) and (b) life-years lost.

The five remaining selected articles mostly resulted from the string “cost and Mediterranean diet” and all articles dealt with micro-costing analysis, as well as with direct and indirect cost evaluation: two papers used a cross-sectional study [32,36]; another two were cohort surveys [34,35] and one other, a narrative review [33]. Lopez *et al*. [35] examined the costs of observed dietary patterns in a Mediterranean Spanish cohort evaluated by a food frequency questionnaire. People that adhered to a Mediterranean dietary pattern, with a higher score of adherence, were positively associated with increased costs of daily food consumption after adjusting for age and sex, whereas a higher score on the Western dietary pattern was inversely associated with cost.

The other resulting cohort survey was carried out by Goulet *et al*. [34]. The purpose of this study was to evaluate the impact of adopting a Mediterranean diet on dietary cost and energy density in free-living conditions. Total daily dietary cost was calculated using a price list, including all items from the FFQ. Results suggested that the adherence to a nutritional intervention program promoting the Mediterranean food pattern is not associated with increased daily dietary cost or energy cost, but led to a reduction in energy density. In a cross-sectional survey, Schroder *et al*. [34] determined the cost differences between low and high adherence to two dietary patterns, like the Mediterranean diet or diets closely adhering to the Healthy Eating Index (HEI), which have been inversely associated with body mass index (BMI) and obesity. Monetary costs for all 165 food items of the food frequency questionnaire (FFQ) administered to the subjects were calculated by multiplying the amount of food consumed from the FFQ with the average national price. The main results of the study were that monetary diet costs increased with higher adherence to the Mediterranean Diet Score (MDS) and HEI. Furthermore, both dietary patterns were inversely associated with BMI and obesity after controlling for several confounders. In the other cross-sectional study, Vlismas *et al*. [32] calculated the current cost of the Mediterranean diet in Greece, measured on common Greek dietary choices through a semi-quantitative FFQ, and evaluated the role of diet cost in the development of cardiovascular events after a five-year follow-up. The Mediterranean Diet Score (MedDietScore) was applied to assess overall adherence to this pattern using scores of eleven food variables and alcohol, according to the principles of the Mediterranean diet. They found that diet cost was correlated marginally to MedDietScore. No significant association was found between diet cost and five-year incidence of CVD. However, adherence to the traditional Mediterranean diet was inversely associated with the development of CVD after adjustment for various potential confounders, including diet cost. They concluded that quality, but not cost of the diet is associated with the development of CVD. Finally, the last article resulted is a narrative review in which Drewnowski *et al*. [33] reported that some nutrient-rich low-energy-density foods associated with the Mediterranean diet were expensive, though others that also fit within the Mediterranean dietary pattern were not. The precise balance between good nutrition, affordability and acceptable social norms is an area that deserves further study. The new Mediterranean diet can be a valuable tool in helping to stem the global obesity epidemic.

The population and the countries considered in the studies were different: one analyses was conducted in Australia, one in France, two in Greece, two in Spain, one in the USA and one in Quebec City (QC, Canada). Two studies of the cost-effectiveness analysis type had a societal perspective (the two reviews) [28,30], but in the study carried out by Panagiotakos *et al*. [28,30], the perspective was not declared.

All the extracted data and the main results of the studies concerning the Mediterranean diet costs are shown in Table 2, while data and results of the cost-effectiveness analysis are shown in Table 3.

### 3.3. Quality Assessment

Drummond’s checklist, as modified by La Torre *et al.* [27], the weighted median score was used in order to assess the quality of the economic evaluation on Mediterranean diet studies. The articles underlying quality assessment were only of the cost-effectiveness analysis study type. Therefore, the three articles [25,26,27] were reviewed with the assigned score quality, as shown in Table 1.

The maximum score quality of 118/118 (100%) was obtained by Dalziel *et al*. [30], and the lowest score, 73/118 (61.86%) was assigned to Panagiotakos D. *et al*., 2007 [29]. An in-between score of 93/118 (78.81%) was assigned to Dalziel *et al*., 2006 [30]. The mean score quality for all three articles considered was high (80.2%).

## 4. Discussion

Due to the global epidemic of several chronic diseases, which, among others, are caused by obesity, the adoption of healthy eating patterns has been at the center of interest in many studies [12,38,39,40]. In this context, the Mediterranean diet is considered a benefit to human health in general, in terms of both primary and secondary prevention of CVD and other chronic diseases.

It is well known that the incidence rates for cardiovascular diseases show a high geographical variability: regions considered, from the Mediterranean Basin, with respect to Northern Europe and the USA, have a lower incidence of cardiovascular diseases (CHD). Advice on how to adopt a Mediterranean diet was shown to induce behavior change in patients after first myocardial infarction [37], as confirmed by a change in nutrient intake consistent with the dietary recommendations. The diet was also shown to be effective in preventing further cardiac events and reducing mortality [41].

The present review of the scientific literature underlines the cost-effectiveness of the adherence to the Mediterranean diet as a prevention strategy against mortality and morbidity due to degenerative pathologies if compared to a non-Mediterranean diet and investigates the impact of adopting a Mediterranean diet based on dietary cost. The analyzed studies show that the Mediterranean diet has the potential to modify disease outcomes and the costs of managing them. The results of the previously published studies are summarized in Table 2 and Table 3.

The first part of our review took into account the cost-effectiveness of the Mediterranean diet: Dalziel and colleagues in 2006 carried on an evaluation aimed at assessing the economic performance of the Mediterranean diet for patients after first acute myocardial infarction (AMI) [28]. The sample consisted of 605 patients aged >70 years who had survived a myocardial infarction within six months of enrolment. The Mediterranean diet, compared with a prudent Western diet, was estimated to cost AU $1013 (U.S. $703, €579) per QALY gained per person. There was a mean gain in life years of 0.31/person and a gain in quality-adjusted life years of 0.40/person. According to this study, the Mediterranean diet is highly cost-effective for persons after first AMI and represents an exceptional return on investment.

Panagiotakos *et al*. [29] performed a cross-sectional study on a sample of 3042 healthy adults and an economic analysis to assess the relationship between the Mediterranean dietary pattern with the 10-year risk of developing CHD compared to a non-Mediterranean diet, as well as its economic consequences. Total healthcare cost was estimated to be €336.720 in those who were “away” and €35.880 in those who were closer to this diet pattern. Life-years lost due to disability were estimated to be 6.8 in those who were “away” and 0.9 in those who were “close” to this diet pattern. The incremental cost-effectiveness ratio was €50.989.

The study conducted by Dalziel and Segal [30] evaluated the economic performance of 10 nutrition interventions: Mediterranean diet, intensive lifestyle change (nutrition and physical activity) to prevent diabetes, reduced fat diet for persons with Impaired Glucose Tolerance (IGT), nutritional counseling in general practice (GP), nurse counseling in GP, Oxcheck Health Checks in GP, Gutbusters Workplace (for men),Talking Computer, Multi Media 2 fruit 5 veg Campaign and the FFFF (Fighting Fit, Fighting Fat) Media Campaign.

In relation to major disease outcomes, the Mediterranean diet had cost-effectiveness ratios of AU $3300 (U.S. $2500, £1300) per non-fatal AMI averted and AU $5300 (U.S. $4000, £2100) per death averted. Mediterranean diet intervention for persons after AMI and intensive lifestyle change to prevent diabetes performed exceptionally well: AU $1020 (U.S. $760, £410) and AU $1880 (U.S. $1410, £750) per QALY gained, respectively. Some interventions, notably the Mediterranean diet and the two interventions to prevent type 2 diabetes, as well as Gutbusters Workplace, are cost-saving under plausible sets of assumptions. The Mediterranean diet and intensive lifestyle change to prevent diabetes are the most certain and cost-effective interventions based on good quality trials.

The last part of our review concerned the cost of individual food items of the Mediterranean diet, since dietary cost plays an important role in food choice.

The ATTICA study [32] calculated the cost of the Mediterranean diet in Greece and evaluated the role of diet cost in the development of cardiovascular events after a five-year follow-up. The cost of the diet was measured in €/week based on common Greek dietary choices, while baseline dietary habits were assessed through a semi-quantitative FFQ. According to this study, the weekly cost of participants’ diets varied from 5.35 to 83.57 €/week in men (mean 25.45 (SD 6.80) €/week) and from 10.89 to 55.49 €/week in women (mean 25.63 (SD 6.30) €/week).

Drewnowski and colleagues tested the viability of the Mediterranean diet as an affordable low-energy-density model for dietary change [33]. The results of their study reinforce the point that Mediterranean-style foods can be obtained at all price ranges, whether calculated per 100 g or per 4.18 MJ (1000 kcal). The only condition is to include more grains, legumes, nuts, vegetables and fruit and less leafy greens and fresh fish; doing so allows re-creation of the Mediterranean eating pattern at an affordable cost.

Goulet *et al*. [34] suggested a North American version of the Mediterranean diet; diets for low income consumers, for whom food price is a key factor determining food choice, may be high in sugar and fat simply because these are the cheapest source of dietary energy available. Adherence to the Mediterranean diet led to increased cost related to vegetables, fruits, legumes, nuts and seeds, canola/olive oil, whole grains, poultry and fish (*p* ≤ 0.01) and to reduced dietary cost for red meat, refined grains, desserts and sweets and fast food (*p* ≤ 0.008). In conclusion, the Mediterranean food pattern is not associated with increased daily dietary cost or energy costs, but leads to a reduction in energy density. Consequently, increased costs should not be considered as a barrier to the promotion and adoption of a Mediterranean diet.

A Spanish cohort study [35] showed that a healthy Mediterranean dietary pattern is more expensive (fifth quintile *vs*. first quintile of scores of adherence: +€0.71 (+$0.90) (95% CI: +€0.67 to +€0.74) per 1000 kcal) than a Westernized dietary pattern (fifth quintile *vs*. first quintile of scores of adherence: −€0.64 (2 $0.80) per 1000 kcal (95% CI: −€0.68 to −€0.61) after adjusting for potential confounders among healthy middle-aged Spanish graduates. After adjusting for dietary pattern scores and other potential confounders, higher daily food costs were significantly associated with greater weight gain. The Mediterranean dietary pattern is more expensive to follow than a Western dietary pattern. This economic barrier should be considered when counseling patients about following a healthy diet, because cost may be a prohibitive factor.

Finally, another Spanish cross-sectional survey [36] analyzed the monetary cost of the adherence to the traditional Mediterranean diet and the Healthy Eating Index (HEI). Data showed that monetary diet costs increased with higher adherence to the Mediterranean Diet Score (MDS) and the HEI, both inversely associated with BMI and obesity.

Developing nations undergoing nutrition transition also replace the traditional plant-based diets with more simple sugars and more added fats. Such energy-dense foods, high in fats, sugars and sodium, have the advantage of being good-tasting, affordable and convenient. Providing dietary energy at very low cost, they are preferentially consumed by lower income groups. The new diets tend to be energy-dense, supplying more energy, but fewer nutrients per gram [24,42].

It has been demonstrated that education is the strongest determinant of socioeconomic differences in food habits [35,43].

Further, food choice is strongly influenced through economic constraints. In addition, there will always be variation in the cost of food between different regions of the country, seasons of the year and types of establishment the food is purchased from [35].

It is well known that there are considerable challenges in estimating food costs: in fact, the same items can vary considerably depending on volume, quality, where they are purchased, brand, *etc*. Economic constraints and their effects on food choices have to be taken into account for the development of dietary tools aimed at weight gain prevention. Limited economic resources might be a stronger criterion for food choice in low-income groups than health concerns. This might be of importance for public health policies in an effort to develop strategies aimed at promoting healthy diets that prevent weight gain.

Anyway, Mediterranean dietary patterns are flexible and adjustable according to specific needs and preferences, because of different economic and ethno-cultural settings, and should be adopted by individuals with a long-term perspective who are actively looking for ways to improve their health.

Healthy dietary recommendations are also provided as official guidelines by numerous medical and governmental institutions. Assessment, interventions and changes are possible with integrated approaches, which are more effective if respectful of individuals and different cultures. Assessment tools and integrated interventional strategies are available, but the widespread knowledge, skills and competence of well-trained individual medical doctors is still lacking.

The main limitations of this work relate to the scarcity of studies evaluating the relationship between food costs and adherence to different food patterns. In addition, in the selected studies, the score for the adherence to the Mediterranean diet was calculated in different ways and through several types of food frequency questionnaires. Furthermore, the results of the selected studies are only partially comparable, because of different geographical settings, dissimilar characteristics of the participants, *etc*. The very small number of reported economic studies is of concern, given the importance of nutrition as a risk factor for the incidence and progression of common chronic diseases and obesity. Another limitation concerns the quality of the studies and the data that support modeling the relationships between intermediate outcomes and health. It is desirable that interventions are compared with all other likely and available options for care in order to assess the true opportunity of the intervention in terms of cost.

## 5. Conclusions

To our knowledge, this is the first complete economic evaluation accomplished, assessing both micro-costing analysis and including direct and indirect costs of the adherence to the Mediterranean dietary pattern and its economic performance in terms of cost-effectiveness as a prevention strategy for degenerative pathologies.

There is a substantial body of evidence linking the Mediterranean diet especially to cardiovascular risk reduction and prevention. Despite this, Mediterranean societies are rapidly withdrawing from this eating pattern, orienting their food choices toward products typical of the Western diet patterns. Possible causes of this phenomenon could be the increasing prices of some of the main food items of the Mediterranean pyramid; this seems to have led people to give up this eating pattern in favor of less expensive products that allow people to save money, but that are unhealthy. Many studies suggest that diet quality follows a socio-economic gradient highlighting how disadvantaged people present higher rates of obesity, diabetes, cardiovascular disease and some types of cancer, showing a linear relationship between food cost and adherence to eating patterns and obesity.

There is the need to conduct more homogeneous studies that evaluate the adherence to the Mediterranean diet and include a standardized and validated, interviewer-administered semi-quantitative food frequency questionnaire at enrolment (that contain the same foods and beverages with standardized sizes) to calculate a Mediterranean diet score reflecting adherence to the traditional Mediterranean diet. Standardized and homogeneous studies, preferably longitudinal study designs, and trials could be useful to evaluate the real benefits of the Mediterranean diet and the true role it plays in the prevention of degenerative pathologies, in the improvement of life expectancy, in net health gain, and in the reduction of total lifetime costs.

Prevention policies should consider the economic barriers associated with following a healthy diet, because cost may be a prohibitive factor.

There has been widespread and growing interest in health economic analyses, and the interest in the quality of economic analyses is increasing in the field of decision making. At the same time, there is emerging need for assuring methodological and reporting quality. This could improve critical evaluations of the literature and push them to perform better on cost-effectiveness or cost-utility analyses in future research. With this in mind, Drummond’s checklist is recommended as a useful tool, as was evidenced in our study [22,23].

Anyway, since several and complex variables exist in driving diet choices, additional research to explore reasons for diet choices is needed.
